# Genetic Engineering of Dystroglycan in Animal Models of Muscular Dystrophy

**DOI:** 10.1155/2015/635792

**Published:** 2015-08-24

**Authors:** Francesca Sciandra, Maria Giulia Bigotti, Bruno Giardina, Manuela Bozzi, Andrea Brancaccio

**Affiliations:** ^1^Istituto di Chimica del Riconoscimento Molecolare, CNR c/o Istituto di Biochimica e Biochimica Clinica, Università Cattolica del Sacro Cuore, 00168 Roma, Italy; ^2^School of Biochemistry, Bristol University, Bristol B58 1TD, UK; ^3^Istituto di Biochimica e Biochimica Clinica, Università Cattolica del Sacro Cuore, Roma, Italy

## Abstract

In skeletal muscle, dystroglycan (DG) is the central component of the dystrophin-glycoprotein complex (DGC), a multimeric protein complex that ensures a strong mechanical link between the extracellular matrix and the cytoskeleton. Several muscular dystrophies arise from mutations hitting most of the components of the DGC. Mutations within the DG gene (*DAG1*) have been recently associated with two forms of muscular dystrophy, one displaying a milder and one a more severe phenotype. This review focuses specifically on the animal (murine and others) model systems that have been developed with the aim of directly engineering *DAG1* in order to study the DG function in skeletal muscle as well as in other tissues. In the last years, conditional animal models overcoming the embryonic lethality of the DG knock-out in mouse have been generated and helped clarifying the crucial role of DG in skeletal muscle, while an increasing number of studies on knock-in mice are aimed at understanding the contribution of single amino acids to the stability of DG and to the possible development of muscular dystrophy.

## 1. Introduction

The extracellular matrix receptor dystroglycan (DG) is highly expressed in skeletal muscle and in several developing and adult tissues, typically in cell types that adjoin basement membranes, such as epithelial and neural tissues [1–3].

DG is composed of two subunits, *α*- and *β*-DG, deriving from a posttranslational cleavage of a single mRNA species encoded by a single gene (*DAG1*) [4]. *α*-DG is an extracellular protein characterized by an extensive and heterogeneous glycosylation mainly concentrated within an elongated central mucin-like region which separates two globular domains, the N- and C-terminal domains [5]. *α*-DG binds with high affinities to the LG domains-containing extracellular proteins, such as laminin-*α*2, perlecan, and agrin, and in turn interacts noncovalently with the *β*-subunit, a transmembrane protein [6]. The cytosolic domain of *β*-DG is anchored to actin through the interaction with dystrophin [7–9], and *β*-DG also constitutes a scaffold for proteins involved in signal transduction such as Gbr2 and ERK [10, 11].

**Figure 1:**
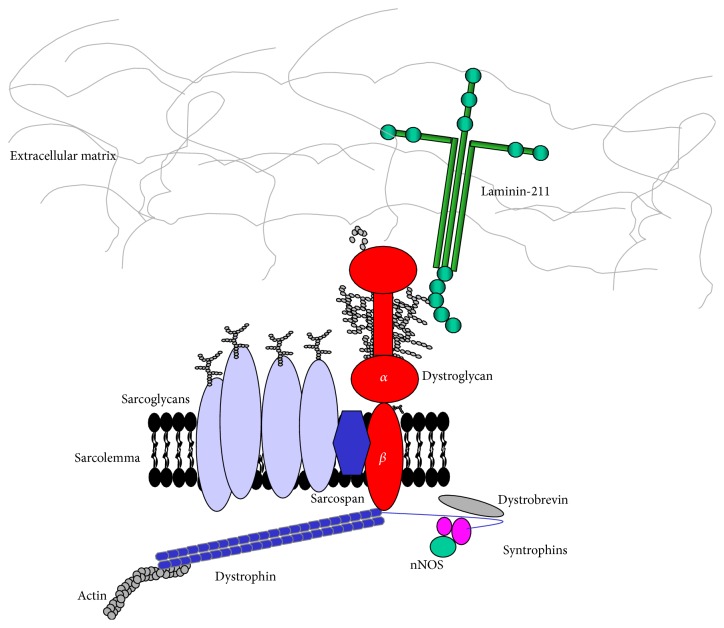
Schematic representation of the dystrophin-glycoprotein complex (DGC) in skeletal muscle. This multiprotein complex anchors the extracellular matrix (ECM) to actin and other components of the cytoskeleton. O-Mannosylated *α*-DG is a central component of this complex and serves as binding partner for a number of ECM proteins containing LG domains, such as laminin-211. *β*-DG is a transmembrane protein and binds the actin cytoskeleton* via* the direct interaction with dystrophin. Other intracellular molecules being a part of, or associated with, DGC are dystrobrevin, syntrophins, and neural nitric oxide synthase (nNOS).

In skeletal muscle, DG is the central component of the dystrophin-glycoprotein complex (DGC), a multisubunit protein complex which links the actin cytoskeleton to the extracellular matrix [12] (Figure 1). Other members of the DGC include transmembrane proteins such as sarcoglycans and sarcospan and multiple cytoplasmic proteins, including dystrobrevin and syntrophins.

The role of the DGC in muscle is to provide mechanical reinforcement to the sarcolemma and to maintain membrane integrity during cycles of contraction and relaxation. In fact, mutations in any components of the DGC cause distinct forms of muscular dystrophy [13]. In humans, mutations in dystrophin lead to Duchenne and Becker muscular dystrophy [14], mutations in sarcoglycans cause limb-girdle muscular dystrophy [15–19], and mutations in laminin-*α*2 cause congenital muscular dystrophy [20]. Recently, mutations in* DAG1* have been reported in three patients, affecting DG function by impairing glycosylation of *α*-DG or by presumably disrupting the *α*/*β*-DG binding interface [21–23].

Moreover, several mutations in 12 proteins involved in the O-mannosyl-glycosylation pathway of *α*-DG have been identified so far which lead to a variety of clinical symptoms, including severe muscular dystrophy and abnormal central nervous system development and function. These diseases are defined as “secondary dystroglycanopathies” (for recent reviews see [24, 25]). The defective O-mannosyl glycosylation of *α*-DG impairs its multiple interactions with its extracellular partners, eventually destabilizing the link between the cytoskeleton and the extracellular matrix. Secondary loss of *α*- and *β*-DG at the muscle membrane also occurs in Duchenne and Becker muscular dystrophies [26] and in some forms of limb-girdle muscular dystrophy [18].

The recovery of DG glycosylation state* via* transgenic overexpression of LARGE, a putative enzyme involved in the first steps of the posttranslational processing of *α*-DG, has been proposed as a therapeutic strategy for muscular dystrophy [27], although with conflicting outcomes [28–30].

The recently emerging data on patients affected by primary and secondary dystroglycanopathies reinforce the notion that a correct expression and modification of DG are crucial for muscle fibres stability and function. A relevant amount of genetic engineering work has been carried out so far on the* DAG1* gene in several laboratories. This review will be focused on the animal models generated to understand the function of DG in skeletal muscle, as well as in other tissues, and to better understand its involvement in neuromuscular disorders (Tables 1 and 2).

## 2. From Knock-Out Mice to the Different Strategies to Circumvent Embryonic Lethality

In 1997 the DG knock-out mouse was generated and analyzed in Kevin Campbell's Laboratory [31]. The targeting vector was designed to replace a portion of the* DAG1* second coding exon with the neo-cassette following homologous recombination.* DAG1*-null allele resulted in a deletion in the exon including the 3′ splice acceptor site and a large portion of the coding sequence of *α*-DG. Animals that were heterozygous for the targeted allele appeared healthy and bred normally. Interestingly, DG transcripts in skeletal muscle of heterozygous mice were only 10–20% lower than those in wild-type mice, suggesting a compensatory increase in the expression level of the untargeted allele. Accordingly, DG protein levels in skeletal muscle were also comparable between wild-type and heterozygous animals. However, the DG knock-out was lethal for homozygous mice embryos that died at the embryonic day 6.5 because of the disorganization of Reichert's membrane, one of the first specialized extraembryonic basement membranes. The absence of laminin receptor precluded the assembly of laminin in a network and the distribution of laminin and collagen-IV appeared patchy, suggesting a crucial role of DG in the organization of the basement membranes [31]. This conclusion was further confirmed by the molecular analyses of the embryoid bodies derived from homozygous* DAG1*-null ES cells in which an ordered basement membrane failed to form [32].

As DG is involved in the development of basement membranes, it certainly is fundamental for normal human development, and the failure to identify null mutations in* DAG1* linked to muscular dystrophies in humans is probably due to early embryonic lethality of such mutations. Interestingly, Frost et al. described a patient affected by a mild myopathy with central nervous system involvement who was heterozygous for a DNA deletion which included also the DG gene [33]. In this patient, only 50–60% of native DG is produced and correctly glycosylated thus showing a much lower degree of compensation compared to the heterozygous DG-null mouse. Although other genes present in the same deleted region could account for the phenotype, this case report suggests the possibility that the heterozygosis for DG-null mutations (haploinsufficiency) could produce pathological consequences in humans. A substantial genetic screening effort, carried out on an enlarged number of patients, would be necessary for the identification of additional cases that may be related to the haploinsufficiency of DG.

To circumvent the embryonic lethality of the DG knock-out mouse, highly chimaeric mice, generated with ES cells targeted for both* DAG1* alleles, were generated [34]. In chimaeric mice deficient in DG rescued from the embryonic lethality, skeletal muscle differentiated normally but they developed a progressive muscular dystrophy reminiscent in many respects of that of mice with double mutations in dystrophin and utrophin [35]. Significant differences in fibre size, central nuclei, and connective tissue infiltration characterized the skeletal muscle histology of DG-null chimaeric mice that die at 13 months [34]. DG plays a crucial role also in stabilizing acetylcholine receptors [36] and consequently in chimaeric mice the neuromuscular junctions (NMJs) were grossly disorganized and disrupted [34]. In the most severely affected mice, the heart appeared dilated and with an extensive connective tissue hyperplasia. At the sarcolemma of DG-null chimaeric mice the entire DGC complex was disassembled, with dystrophin and sarcoglycans absent in many fibres. However, laminin-*α*2, perlecan, and agrin were expressed at wild-type levels and the basement membrane appeared organized in an ordered network. It is likely that, in differentiated skeletal muscle, the expression of integrins or other extracellular matrix receptors exert an important compensatory effect in supporting the skeletal muscle differentiation and basement membrane assembly [37]. However, the DG-null chimaeric mice pointed out the central role of DG in the maintenance of the DGC and muscle integrity.

An additional step forward in understanding the functional role of DG in skeletal muscle came from the conditional inactivation of skeletal muscle DG using the Cre-loxP system under the muscle creatine kinase (MCK) promoter [38]. The MCK-Cre/DG-null mice were viable and born with the expected frequency; they developed muscular dystrophy around 4–6 weeks of age but the phenotype became milder with advanced age. As a matter of fact, satellite cells, which had not been targeted by the Cre recombinase, supported the muscle regeneration and formation of novel fibres expressing DG and the other components of DGC. Moreover, in old mice, muscle fibres appeared hypertrophic and larger, as compared with controls and with the other mouse models of muscular dystrophy. Like in chimaeric DG-null mice, also in MCK-Cre/DG-null mice, laminin-*α*2 was expressed and the basement membrane was correctly assembled. However, while in chimaeric DG-null mice the NMJs were disrupted, in MCK-DG-null mice they were preserved.

In MORE-DG-null mice [38], the inactivation of* DAG1* was driven by Cre recombinase under the control of the Mox 2 promoter enabling the targeting of DG in all tissues of the embryo, while DG was still expressed in extraembryonic membranes to circumvent embryonic lethality. MORE-DG-null mice were significantly smaller than control littermates, a majority of the mice died within 48 h after birth, and the remaining mice typically failed to survive the fourth postnatal week. In addition, MORE-DG-null mice exhibited profound muscle weakness and muscular dystrophy was present at birth, reminiscent of a secondary dystroglycanopathy phenotype (see next paragraph) [39]. Consistent with the results obtained with MCK-Cre/DG-null mice, MORE-DG-null mice displayed severe impaired regeneration capacity since satellite cells were also targeted by Cre recombinase under the control of the Mox 2 promoter [38].

The phenotype observed in chimaeric and conditional knock-out mice demonstrated the importance of DG for the stability of the DGC and for the structural integrity of the sarcolemma. However, the overexpression of DG in transgenic mice onto an* mdx* background did not inhibit muscular dystrophy; on the contrary, it exacerbated the phenotype by decreasing the utrophin and sarcoglycans expression at the sarcolemma [40].

## 3. Conditional DG Knock-Out in the Brain Recapitulates the Outcome of Secondary Dystroglycanopathies

Fukuyama congenital muscular dystrophy (FCMD), muscle-eye-brain disease (MEB), and Walker-Warburg syndrome (WWS) are congenital muscular dystrophies (CMDs) with associated developmental brain defects [41–43]. The genes that are mutated in these disorders are those of the enzymes involved in the O-mannosyl glycosylation of *α*-DG, in particular protein-O-mannosyl transferase 1 (POMT1), protein-O-mannosyl transferase 2 (POMT2), protein-O-linked mannose beta 1,2-N-acetylglucosaminyltransferase (POMGnT1), and fukutin, an enzyme indirectly implicated in a pathway to further modify the phosphorylated O-linked mannose located in the mucin-like domain of *α*-DG [44].

The conditional DG knock-out mouse in the brain was produced, using the Cre-LoxP methodology, in order to analyse the function of DG in the central nervous system and to demonstrate the role of DG in the brain malformations seen in CMDs [45]. Brain-selective expression of Cre recombinase was accomplished using a human glial fibrillary acid protein (GFAP) promoter expressed as early as embryonic day 13.5. GFAP-Cre/DG-null mice followed the expected Mendelian distribution and were fertile.

In the GFAP-Cre/DG-null mice cerebral cortex, DG was not expressed in the astrocytes abutting the brain surface (glia limitans) and cerebral microvessels, in radial glia and in a subset of neurons that are the progeny of radial glia [45, 46]. Also the localization of dystrophin isoforms was impaired in these cells. The basal lamina of the glia limitans that plays a critical role for normal cortical development was severely disrupted. The results were a number of brain structural developmental defects similar to those seen in MEB, WWS, and FCMD patients. The abnormalities of the glia limitans permitted the overextended migration of neurons in the developing brain, which is the most important diagnostic feature of cobblestone lissencephaly observed in the most severe cases of secondary dystroglycanopathy. Such mice also lacked the usual fissure between the brain's hemispheres, a characteristic of WWS, and suffered from an overabundance of glia.

Despite the large similarities with CMDs, GFAP-Cre/DG-null mice did not recapitulate the most severe characteristics observed in the brain of patients affected by WWS. On the contrary, the earlier suppression of DG expression in MORE-DG-null mice (in which Cre recombinase under the control of the Mox 2 promoter operates at E7.5) was sufficient to cause malformations that broadly resembled the clinical spectrum of WWS, including hydrocephalus and ocular malformations with structural defects of both the anterior and posterior chambers of the eye [39].

The* DAG1* gene was targeted to generate other mouse models for the detailed analysis of the role of DG in the central nervous system and in the visual function. For example, the following two novel DG mouse models were created and analyzed by Satz and colleagues [47]: (1) the conditional knock-out nestin-CRE/DG-null mouse, in which Cre recombinase was under the control of the rat nestin enhancer expressed in neuroepithelial precursor cells and in the retina as early as embryonic day 9.5 (further analysed in [48, 49]) and (2) a knock-in mouse expressing a truncated DG lacking the entire *β*-DG cytodomain (in which a premature stop codon was inserted after Lys778 resulting in the presence of only 4 amino acids in the cytodomain of *β*-DG). Surprisingly, according to the authors, a reasonable number of knock-in mice were obtained [47]. It remains unclear whether these mice would display some pathologic effect in their skeletal muscle.

Moreover, Omori and colleagues developed a retinal photoreceptor-specific DG conditional knock-out (Crx-Cre/DG-null) mice, in order to analyze the role of DG localized at the presynaptic elements of photoreceptor cells [50]. The results showed the crucial role of presynaptic DG for both the formation of proper photoreceptor ribbon synaptic structures and normal retinal electrophysiology.

## 4. DG Knocking-In: A Powerful Tool to Dissect the Role Played by Specific Amino Acids

### 4.1. DG^T190M^/DG^T190M^, the First Case of Primary Dystroglycanopathy

The first case of a homozygous missense mutation in the* DAG1* gene was described in a 16-year-old patient [21] originally described as affected by a mild form of limb-girdle muscular dystrophy associated with mental retardation but normal brain imaging (recently classified as limb-girdle muscular dystrophy 2P [51]). Immunofluorescence and immunoblot of muscle biopsies showed that *α*-DG was hypoglycosylated and had a reduced affinity toward laminin. The mutation (T192M), located within the RNA binding protein-like domain of the *α*-DG N-terminus [52], is supposed to reduce,* via* a mechanism that needs to be fully elucidated yet, the binding between *α*-DG and the glycosyltransferase LARGE, an interaction that is essential for the posttranslational modification of *α*-DG and for the DG's laminin-binding activity [53]. Recently, a mild form of muscular dystrophy characterized by hypoglycosylated *α*-DG was associated with the compound heterozygous missense mutations (V74I and D111N) with both mutated sites located within the N-terminal domain of *α*-DG and a pathological molecular mechanism similar to the one described for the T192M mutation was hypothesized [22].

A knock-in mouse was generated introducing the mutation T192M (that in mouse corresponds to T190M) by homologous recombination [21]. Heterozygous mice were normal, while homozygous knock-in mice presented abnormalities consistent with those observed in the patient. Analysis of muscle biopsies revealed hallmarks of muscular dystrophy, such as centrally nucleated fibres, and a hypoglycosylated *α*-DG with a decreased laminin-binding activity. The T190M mutation interfered also with the organization of the NMJ. Although no structural abnormality was evident in the brains of the knock-in mice, these mice had abnormal hind limb clasping, a phenotype common to mouse models featuring neurologic impairment. Interestingly, in the heart, the mutated *α*-DG still bound to laminin and no obvious signs of any pathological abnormality were observed.

Recently, a novel homozygous mutation in* DAG1* has been identified in a Libyan family with two siblings affected by a dystroglycanopathy resembling a MEB-like condition [23]. The mutation (C669F) hits an amino acid already identified as an important site for the interaction between *α*- and *β*-DG and for the overall stability of the complex, but the knock-in mouse model has not been generated yet [54, 55].

### 4.2. DG^Y890F/Y890F^/mdx

Although not directly linked to a specific disease, a knock-in mouse hitting a tyrosine within the cytodomain of *β*-DG represented a tool for dissecting the role of the phosphorylation of DG in the skeletal muscle and for testing novel therapeutic ideas [56].

In fact, previous studies suggested that tyrosine phosphorylation of DG at the site Y892 may be an important mechanism for modulating the association of DG with its cellular binding partners dystrophin and utrophin and may also work as a signal for proteasome degradation of DG [57–59].

The knock-in mouse DG^Y890F/Y890F^ was generated using homologous recombination in ES cells [56]. Both heterozygous and homozygous DG^Y890F/Y890F^ mice appeared normal and healthy. Skeletal muscle analysis of knock-in mice did not reveal any differences or abnormalities compared to the wild-type mice.

In order to assess whether the inhibition of tyrosine phosphorylation in DG had any beneficial effect on dystrophic skeletal muscle, DG^Y890F/Y890F^/*mdx* mice were further generated [56]. Interestingly, the expression of Y890F mutant DG in an mdx background significantly improved the muscle phenotype, reducing the number of centrally nucleated fibres and the levels of creatine kinase. Moreover, also an improvement in resistance to eccentric contraction-induced injury was observed in DG^Y890F/Y890F^/*mdx* mice. Changing a single phosphorylation site in DG reinforced the DGC sarcolemma localization preventing the proteasome degradation of DG. In fact, the inhibition of tyrosine phosphorylation of DG in mdx mice was sufficient to restore the sarcolemma localization of DG, *α*-sarcoglycan, and sarcospan also in the absence of dystrophin, while utrophin was confined at the NMJ [56]. Moreover, an increase in the plectin expression/localization at the sarcolemma was also observed. Plectin is a cytolinker protein that binds *β*-DG at different sites, thus providing a stabilizing link between DG and the cytoskeleton [60]. It was already known that treatment with proteasomal inhibitors improves the muscle pathophysiology in some mouse models, such as* mdx* mice [61] or *dy* ^3*K*^ mice [62], and in this context the DG^Y890F/Y890F^/*mdx* mouse highlights novel targets for therapeutic intervention.

## 5. Mice Overexpressing DG

The overexpression of multiple and randomly integrated copies of the coding sequence of DG was obtained by microinjection of a pBS-HSAvpA cDNA construct into fertilized CB6 oocytes. Mice overexpressing wild-type DG were normal compared to control [63]. Interestingly, transgenic lines overexpressing DG mutated in the cleavage site S654 were also created in order to understand the role of the posttranslational cleavage resulting in the production of the two interacting subunits [63]. In the transgenic DG^S654A^ mice only the uncleaved DG precursor was correctly expressed, while the expression of endogenous and processed DG was inhibited. In DG^S654A^ mice, most muscles were dystrophic with increased levels of central nuclei. The lack of the DG cleavage and the presence of muscular dystrophy correlate with altered glycosylation of *α*-DG. In addition, the expression of dystrophin and *α*-sarcoglycan decreased, while utrophin and laminin-*α*5 were upregulated, probably as a secondary effect of muscle regeneration. Aberrant NMJs were observed in DG^S654A^ mice, although also DG^WT^ mice occasionally showed fragmented NMJ [63].

## 6. Conditional DG Knock-Out Mice in Other Tissues Compared to Skeletal Muscle

DG is also highly expressed in epithelia and in the peripheral nervous system (Table 2). To study the role of DG in the kidney, different conditional knock-out mice were created to selectively delete DG from podocytes, ureteric bud, metanephric mesenchyme derivatives, and all renal epithelial cells using the Cre-lox system under the control of podocin, HoxB7, Pax-3, and Pax-2 promoters, respectively [64]. Surprisingly, DG deletion from kidney resulted in no aberrant phenotypes. Kidney formation and function proceeded normally in the absence of DG and the only detectable abnormality was a mild increase of the glomerular basement membrane thickness. This observation was further confirmed in chimaeric mice generated with fukutin-null embryonic stem cells expressing a hypoglycosylated *α*-DG, in which minor glomerular structure abnormalities were found without functional renal defects [65]. These results suggest that DG and its correct glycosylation may be important in the maintenance of podocyte architecture and extracellular matrix assembly; however, the presence of integrin *α*3*β*1 as an additional laminin receptor and basement membrane organizer in podocytes may preserve the structure and functionality of the kidney [65]. Accordingly, there are no reported renal dysfunctions in human patients with impaired DG glycosylation.

To analyse the role of DG in peripheral nerves, DG was disrupted selectively in Schwann cells using the P0 protein promoter and Cre-loxP technology [66]. The loss of DG caused severe neurological dysfunctions, including a slow nerve conduction. In P0-Cre/DG-null mice the myelin sheaths around the nerves were structurally abnormal and extended throughout the internodal segments. The DG-interacting proteins, sarcospan, sarcoglycan, and *α*-dystrobrevin, were lost from the membrane and laminin was not deposited around the nerves. These findings point to the crucial role of DG in myelin integrity and in node of Ranvier structure and function [67].

## 7. Other Animal Models to Study the DG Functions in Muscle and Central Nervous System

DG and most of the members of DGC are highly conserved in vertebrates, including fish, and invertebrates. Therefore, DG offers a wide range of possible animal models aside from mouse to understand the role of DG in the pathogenesis of muscular dystrophies.

### 7.1. *Caenorhabditis elegans*

Interestingly, in* Caenorhabditis elegans,* DG is expressed in epithelial and neuronal tissues but not in muscle. Indeed, a deletion, cg121, that removes most of the coding and some of the 3′ untranslated region of* DGN-1* gene, resulted in viable but sterile animals, with neuronal defects and normal muscles [68].* DGN-1* contains the N-terminal immunoglobulin-like domain of vertebrate *α*-DG and a shorter mucin-like region, while the *α*/*β* proteolytic cleavage region and the residues involved in binding WW and SH3 domain-containing proteins such as dystrophin are all missing [68].

### 7.2. *Drosophila melanogaster*

All known components of the DGC are present in the fruit fly [69]. Several* Drosophila* DG isoforms are generated* via* alternative splicing [70, 71]. Only one of these contains the full mucin-like domain, characterized by significant levels of glycosylation, while DG isoforms that lack the mucin-like domain are required to maintain polarity in the follicular epithelium, suggesting that the isoforms can have different functional roles in* Drosophila* [71]. Moreover,* Drosophila* DG is not cleaved in *α*- and *β*-subunits but it is expressed as a single polypeptide [72].

Interestingly, muscle-specific RNAi-mediated knockdown of DG, as well as of dystrophin isoforms, led to age-dependent, progressive climbing deficits, severe muscle degeneration in adult flies, and defects in neurons migration and eye development [73]. Haines and colleagues, analysing larvae carrying mutant alleles of DG, also established that DG is required in* Drosophila* larval muscles to maintain integrity [74].

The similar defects observed in both flies and humans make* Drosophila* an attractive model for further studies on clarifying the role of the DGC. In particular, using the RNAi knockdown mutants of DG, many genes had been identified as possible regulatory genes of DG and dystrophin, such as genes involved in muscle function and components of Notch, TGF-*β*, and EGFR signalling pathways [75]. Recently, it was also shown that in* Drosophila* the expression level of DG may be buffered in a homeostatic fashion* via* a mechanism mediated by the miR-310s complex which acts directly on the alternative DG 3′-UTR [76]. Deficiencies in the miR-310s complex resulted in cobblestone brain, a phenotype reminiscent of human lissencephaly type II [76]. This evidence represents a seminal result, paving the way for identifying similar regulation mechanisms also in higher vertebrates and mammals.

### 7.3. *Danio rerio*

In recent years,* Danio rerio* (zebrafish) has emerged as a powerful genetic tool to study muscle diseases [77]. Disruption of DG translation using an anti-sense morpholino oligonucleotide (MO) approach, led to the destabilization of the embryos muscle, with loss of sarcomere organisation and necrosis of the developing muscle [78]. The NMJ and central nervous system appeared normal. The lack of the DG protein impaired also the localization of dystrophin. Interestingly, in an ENU (N-ethyl-N-nitrosourea) mutagenesis screen aimed at identifying genes responsible for skeletal muscle disorders, a DG homozygous mutant, patchytail, was found to show impaired locomotion behaviour, dystrophic muscles and ocular and central nervous system defects [79]. The point mutation resulted in a missense amino acid change of valine to aspartic acid (V567D) within the Ig-like domain in the C-terminal region of a-DG, leading to a destabilization and degradation of the protein [79, 80]. The absence of DG led to a reduced expression of dystrophin and laminin-a2. The sarcolemma appeared grossly disorganized and detached from the extracellular matrix. NMJs were normal in patchytail fish, but severe abnormalities of brain and eyes were observed and the embryos did not survive more than 10 days post fertilization. This case represents so far the first case in which a single point mutation is able to induce the complete depletion of DG from tissues, highlighting the importance that even single aminoacids may have for the stability and/or folding pathway of the DG precursor [56, 80]. An additional loss of function mutation was identified in a zebrafish affected by muscular dystrophy (dag1^hu3072^) due to a nonsense mutation (R398>Stop) within the mucin-like region of a-DG, causing the complete loss of DG [81]. The absence of DG led to the dislocation of dystrophin and to muscle fibre detachment, followed by disruption of sarcolemma integrity.

### 7.4. *Xenopus laevis*

The possible DG functions in different tissues of* Xenopus laevis* were analysed using the morpholino knock-out approach. It was shown that, during* Xenopus* development, DG is important for the somitogenesis and that the interaction between DG and the extracellular matrix is indispensable for the alignment of the myoblasts in the somites [82]. The loss of DG influenced the epidermal differentiation in the retinal and renal development [83–85]. Moreover, the injection of rabbit DG RNA into* Xenopus* embryos produced an overexpression of DG that altered the acetylcholine receptors aggregation and the NMJ structure [86].

## 8. Perspectives

Starting from the 1997 knock-out mouse [31], during the last 17 years, an impressive amount of work has been carried out already on DG at the genetic level. It is likely to expect in the next few years a further increase both in the number of patients/families identified who carry mutations specifically within the* DAG1* gene and in the number of animal genetic models that will be generated and analyzed. In particular, it could be particularly interesting to focus on the 3′ region of* DAG1*, corresponding to the *α*/*β*-DG interface, the genetic clinical screenings carried out on still unassigned cases of myopathy presenting with symptoms that may suggest the presence of a dystroglycanopathy. In this specific and very small region (that could be therefore analyzed inexpensively) in fact, mutations have been found to grossly affect the stability and the maturation of the complex, in zebrafish as well as in human patients [23, 79, 81].

*Via* such comparative studies, a full circle will be completed in the elucidation of the function(s) of DG in muscle and nonmuscle tissues. Moreover, the genetic data on human patients and the generation of novel animal models will certainly boost the research on potential therapeutic approaches for human muscular dystrophies.

## Figures and Tables

**Table 1:** DG mouse models characterized by a muscle and/or central nervous system phenotype.

Mouse model	Muscular dystrophy	CNS involvement	NMJs
Chimaeric mice [34]	Progressive	—	Disorganized and disrupted
MCK-Cre/DG-null [38]	Mild	—	Normal
GFAP-Cre/DG-null [45]	—	Neuronal migration errors, brain malformation	—
MORE-DG-null [38, 39]	Severe	Neuronal migration errors, brain malformations, and ocular defects (WWS phenotype)	—
Nestin-Cre/DG-null [47] and Crx-Cre/DG-null [50]	—	Abnormal retinal physiology	—
DG^T192M^/DG^T192M^ [21]	Mild	Some neurological impairments	Compromised
DG^Y890F/Y890F^ [56]	Normal	Normal	Normal
DG^Y890F/Y890F^/mdx [56]	Ameliorated	—	Ameliorated
DG^WT^overexpression [40]	Normal	Normal	25% smaller than normal but only 1% are aberrant
DG^WT^overexpression/mdx [40]	Not ameliorated	—	Not ameliorated
DG^S654A^overexpression [63]	Mild	—	Compromised
DG^Δ*β*cyt/Δ*β*cyt^ [47]	—	Mild effects in the retina	—

—: not analysed.

**Table 2:** Mouse models in which DG was targeted in tissues other than skeletal muscle and brain and additional DG animal models with muscle and central nervous system defects.

Animal model	Phenotype
Kidney specific DG knock-out mouse (podocin-Cre/DG-null, Pax2-Cre/DG-null, Pax3-Cre/DG-null, HoxB7-Cre/DG-null) [64]	Normal
Schwann cells specific DG knock-out mouse (P0-Cre/DG-null) [66]	Severe neurological dysfunctions
DG knock-out in* Caenorhabditis elegans* [68]	Defects in gonad and vulval epithelium and in motoneurons
RNAi knock-out of DG in *Drosophila melanogaster* [73, 74]	Muscle degeneration and neuronal defects
Inhibition of DG translation *via* morpholino antisense in zebrafish [78]	Muscle defects
Zebrafish *patchytail* [79]	Dystrophic muscles, ocular and central nervous system defects
Zebrafish dag1^hu3072^ [81]	Muscular dystrophy
Inhibition of DG translation *via* morpholino antisense in *Xenopus laevis* [82–86]	Defects in the somitogenesis, epidermal differentiation, the retinal and renal developing
Overexpression of DG in *Xenopus laevis* embryos [86]	Aberrant neuromuscular junctions
